# Research on the Impact of AI Introduction on Employee Creativity: The Moderating Role of Technology Overload

**DOI:** 10.3390/bs15101389

**Published:** 2025-10-14

**Authors:** Dexia Zang, Manyu Li

**Affiliations:** Business School, Hohai University, Nanjing 211100, China; 221313040025@hhu.edu.cn

**Keywords:** AI introduction, employee creativity, perceived job autonomy, perceived job feedback, technology overload

## Abstract

The introduction and application of AI are profoundly affecting the working modes and psychological states of employees. For many organisations, AI is not only a tool to improve productivity, but also an important driving force for organisational change. In the context of the rapid development of AI, it is particularly important to explore the impact mechanism of its introduction on employee creativity. This study uses the theory of technological affordances and job characteristics theory to explore the impact of AI introduction on employee creativity. Through an analysis of the questionnaire data of 309 employees, it is found that the introduction of AI has a significant positive impact on employee creativity, and perceived job autonomy and perceived job feedback play a mediating role between the introduction of AI and employee creativity. Technology overload not only negatively moderates the relationship between AI introduction and perceived job autonomy, but also negatively moderates the relationship between AI introduction and perceived job feedback; that is, the higher the technology overload is, the weaker the positive relationship between AI introduction and perceived job autonomy and perceived job feedback is. This study not only provides a new perspective for understanding the opportunities and challenges brought by the application of AI in the workplace, but also provides an empirical basis for enterprises to formulate effective human resource management strategies in the process of digital transformation.

## 1. Introduction

In contemporary society, characterised by the rapid advancement of technology, artificial intelligence (AI) has garnered widespread attention across various sectors as an innovative technology (Dong et al., 2025). The adoption of AI by enterprises is driven by a variety of factors, resulting in its implementation exhibiting significant variation across different industries. Furthermore, the specific AI technologies employed also vary considerably. Recent years have seen a proliferation of globalisation and digitalisation, which have resulted in a significant escalation in the level of competitive pressures faced by enterprises. In order to maintain a competitive advantage in rapidly evolving market conditions, businesses have been compelled to undertake technological upgrades and transformations. This process has progressively integrated AI into all facets of human resource management, including recruitment, training, and performance evaluation (Huang, 2025). As stated in the 2018 research conducted by IBM, its proprietary suite of AI technologies has been demonstrated to provide effective support for personnel recruitment, remuneration planning, staff training, and personalised learning. Indeed, with its widespread societal application, AI-related research has undergone rapid expansion over the past five years, with a predominant focus on economics, consumer behaviour, marketing, human resource management, and corporate strategic management.

Within the fields of corporate management and human resources, the integration and implementation of AI has been shown to exert a significant influence on employees’ working methods and psychological well-being (Han & Huang, 2025). Research into the impact of AI on employee creativity is crucial, with its stimulating mechanisms primarily encompassing three aspects: foundational conditions (such as physical maturity and cultural openness), cultural stability (based on shared values and traditional cohesion), and motivational drivers (originating from intrinsic recognition and the pursuit of wealth) (Kanzola & Petrakis, 2021). As AI technology becomes deeply integrated across industries, its impact on organisational management extends (Amayreh et al., 2025) beyond mere efficiency gains to encompass complex behavioural and cognitive dimensions (Yuan et al., 2025), such as employee creative performance. Of particular note, Cosmina et al. investigated the phenomenon of cognitive debt accumulation when using AI assistants to write papers, a finding that offers valuable insights into how AI influences cognitive processes (Kosmyna et al., 2025). The creativity of employees is identified as a key driver of innovation within organisations (Amabile, 1988). This creativity is dependent on individual cognitive flexibility, the ability to reframe problems, and the process of generating novel and useful ideas. From a process perspective, creativity encompasses a series of socialised cognitive activities, ranging from problem identification and information gathering to idea generation and solution evaluation (Green et al., 2023). The introduction of AI has been demonstrated to exert multidimensional impacts on this process by reshaping task structures, altering cognitive resource allocation, and modifying interaction patterns. Recent studies on the relationship between AI implementation and employee creativity have been found to be inadequate, particularly within the context of China’s unique socio-cultural environment. This has led to a pressing need for further research in this domain. Consequently, the manner in which AI implementation can effectively enhance employee creativity, thereby strengthening organisational innovation capabilities, has become a focal point for corporate managers.

Recent research indicates that AI’s impact on employee creativity is a double-edged sword, with its core mechanism residing in the nature of human–machine collaboration. On the one hand, AI liberates employees’ cognitive resources by handling routine tasks (Dell’Acqua et al., 2023), generative AI can provide abundant inspiration (Haase & Hanel, 2023), and AI systems offer instant feedback to accelerate creative iteration (Jia et al., 2024), significantly boosting creativity. Conversely, excessive reliance on AI may foster cognitive inertia and algorithmic dependency among employees, narrowing their thinking. Perceiving AI as a substitute threat, employees experience diminished creative self-efficacy, thereby inhibiting creativity (S. Zhou et al., 2024). Research indicates that AI can significantly influence employees’ creative thinking and behaviour through multiple mechanisms, yet its effects exhibit considerable complexity and contextual dependence (Boussioux et al., 2024b).

The theory of technological affordances, as proposed by Gibson within the framework of ecological psychology, underscores the potential actions an environment offers to actors (Gibson, 1977). Norman applied this concept to technology, noting that users can interact with their environment in novel ways by understanding and utilising technological capabilities. Within organisational settings, this theory provides a critical lens for explaining how AI influences employee behaviour and cognition, such as autonomy and creativity. The implementation of suitable AI applications has been demonstrated to enhance employees’ sense of control over their work. This is evidenced by an increase in perceived job autonomy, which refers to employees’ perception of task freedom and discretion (Hackman & Oldham, 1976). Additionally, there is a rise in perceived feedback, which encompasses clear information, return on work outcomes, and performance (J. Zhou, 2007). It is hypothesised that these perceptual characteristics play a crucial mediating role between AI implementation and employee creativity. Within this relationship, perceived job autonomy (Hackman & Oldham, 1976) and perceived feedback (J. Zhou, 2007) serve as key mediating variables, reflecting the mechanism by which AI stimulates creativity through granting employees greater control and clearer performance information.

Conversely, excessive technological utilisation has the potential to yield adverse effects. When employees encounter AI systems characterised by information overload, system complexity, or redundant functionality, they may experience technological overload—a psychological state where perceived technological demands exceed one’s processing capacity (Tarafdar et al., 2007). The phenomenon of technological overload has the potential to diminish the positive effects of job autonomy and disrupt the clarity of feedback, thereby inhibiting the unfolding of creative cognitive processes. Consequently, the phenomenon of technological overload (Tarafdar et al., 2007) functions as a pivotal negative moderator, engendering not only the disruption of employees’ perceptions of autonomy and feedback, but also the potential diminution of creative motivation through the augmentation of cognitive load and psychological insecurit (Kellogg et al., 2019).

Against this backdrop, the present study proposes a mediation model grounded in the theory of technological affordances (Gibson, 1977) with a view to systematically examining the mechanisms through which implementation of AI influences creativity in employees. The present study puts forward the hypothesis that the implementation of AI exerts a positive effect on employee creativity via the mediating roles of perceived job autonomy and perceived job feedback. In addition, the study posits that technological overload negatively moderates the impact of AI implementation on perceived job characteristics.

This empirical study focuses on the AI-OB (AI and Organisational Behaviour) domain, establishing a link between the introduction of AI and specific workplace behaviours among employees. This addresses calls from some scholars (Yu et al., 2018) for future research directions and enriches the antecedents of employee creativity. From a practical perspective, the findings suggest that managers should consider more than just the technical deployment of AI when implementing it. They should emphasise harmonising employees’ psychological perceptions with organisational support systems. By providing systematic training and cultural guidance, the negative effects of technological overload can be reduced, thus maximising the positive impact of AI on employee creativity.

## 2. Theoretical Basis and Hypothesis

The theory of technological affordances originated from ecological psychology. Gibson defined affordances as the actionable properties between an object and an actor, that is, what the environment offers the individual, for good or ill (Gibson, 1977). The concept was later adapted to technology studies by researchers such as Donald Norman, who emphasised perceived affordances within human–computer interaction—focusing not only on what actions a technology allows, but also on how those possibilities are recognised by users (Norman, 1999). In the context of digital and AI technologies, affordance theory provides a useful lens through which to understand how characteristics of technological systems enable or constrain specific behaviours, thereby influencing how users—such as employees—interact with and perceive these systems. Its application is especially pertinent in organisational settings, where technology’s affordances can influence perceptions of autonomy, feedback, and creativity, thus rendering it a foundational lens for studying the psychological impact of AI in the workplace.

The core competitiveness of modern enterprises increasingly relies on employees’ creativity and innovative capabilities, while AI can profoundly alter employees’ working environments and cognitive processes, thereby potentially influencing their creativity (Dutta et al., 2025). The theory of technological affordances posits that technology provides individuals and organisations with the potential to leverage their technical capabilities to enhance work performance and gain competitive advantage. Within this framework, AI can stimulate employee creativity by fostering collaboration and providing data-driven decision support. Conversely, excessive reliance on AI may inhibit creative thinking, leading to questioning of professional value and diminished job satisfaction.

To systematically elucidate the underlying mechanisms and boundary conditions through which AI influences employee creativity, this study adopts the theory of technological affordances as its core theoretical framework, integrating the Job Characteristics Model. It clarifies the definition of “availability” within this research and introduces perceived job characteristics. Based on the theoretical framework, it explores the relationship between AI and employee creativity, analysing the mediating role of perceived job autonomy and perceived feedback in this relationship. It focuses on the moderating mechanism of technological overload, examining its boundary role between AI usage and employee behaviour. Finally, integrating these theoretical perspectives, a system of research hypotheses is proposed and a corresponding theoretical model is constructed.

### 2.1. Theory of Technological Affordances

The theory of technological affordances provides an integrative framework for systematically analysing how technology is perceived, interpreted, and utilised within socio-technical systems. This theory focuses not only on the functional attributes of technology itself, but also emphasises the interactions among multiple elements—technology, individuals, organisations, and the environment—particularly prioritising the accessibility, usability, and diffusion mechanisms of new technologies within specific contexts (Leonardi, 2012; Strong et al., 2014). Its core tenet posits that technology offers ‘behavioural possibilities,’ yet whether these possibilities are recognised, accepted, and translated into action by users depends on the combined effects of individual perception, social context, and organisational conditions (Gibson, 1977; Waizenegger et al., 2020).

Within the AI context, this ‘availability’ manifests as behavioural opportunities such as cognitive augmentation, task automation, and real-time feedback (Lu et al., 2013). Specifically, AI offers two key types of affordance: firstly, by automating routine tasks and optimising resource coordination, it enhances employee autonomy and fosters innovation (Leonardi, 2012). Secondly, through data processing and pattern recognition capabilities, it provides real-time performance feedback, enabling employees to adjust strategies, reinforce learning, and stimulate creative behaviour (J. Zhou, 2007). However, the realisation of technological affordances depends not only on technical functionality, but is also moderated by social and individual factors. This aligns with the multi-factor influence perspective emphasised by technology acceptance models (Taherdoost, 2018). For instance, employees’ perceptions of AI usefulness, organisational training, psychological safety, and the complexity and transparency of the technology all influence the actual “realisation” of AI availability (Curtis et al., 2019; Treem & Leonardi, 2013). Introducing this theory facilitates the exploration of mechanisms and boundaries through which AI influences employee creativity, from a ‘technology–person–organisation’ co-construction perspective, providing a theoretical foundation for systematic AI implementation strategies.

### 2.2. Theory of Job Characteristics

Hackman and Oldham proposed the Job Characteristics Theory, which identified five core job attributes: skill variety, task identity, task significance, perceived feedback, and perceived autonomy (Hackman & Oldham, 1976). Perceived feedback refers to whether employees receive clear and explicit feedback on their performance when completing work tasks. Perceived autonomy denotes whether the job affords employees sufficient freedom, independence, and discretion to schedule their work time and determine their working methods. Hackman and Oldham demonstrated and summarised the impact of these five core job characteristics on employee outcomes, including intrinsic motivation, job performance, job satisfaction, turnover rates, and absenteeism rates (Hackman & Oldham, 1976).

Furthermore, Hackman and Oldham proposed that core job characteristics influence work outcomes through three primary psychological states: perceived job meaningfulness, perceived job responsibility, and perceived job outcomes (Hackman & Oldham, 1976). They posited that perceived job meaningfulness is primarily determined by task variety, task consistency, and task significance, while perceived job responsibility is chiefly shaped by perceived autonomy. When job autonomy is high, outcomes depend largely on the employee’s own efforts and decisions, fostering a greater sense of responsibility for task success. Perceived job outcomes are more strongly influenced by perceived feedback. When employees receive more direct and immediate feedback on their performance from the work itself, they develop a more comprehensive understanding of their overall job performance (Hackman & Oldham, 1976). Oldham and Silva also regarded employee creativity as one of the effects of the theory of job characteristics, and proposed that the use of digital equipment and technology in the work situation would improve the work autonomy and work feedback, thus enhancing the work participation and concentration of employees, thus having a positive impact on the proposal and implementation of innovative ideas (Oldham & Silva, 2015). Therefore, this study follows the task-oriented logic of jobs, introduces job characteristics (perceived job autonomy and perceived job feedback), and explores the impact path of AI introduction in the work context on employee creativity.

### 2.3. Introduction of AI and Employee Creativity

According to the theory of technological affordances (Gibson, 1977), the use of specific technologies can provide individuals with action opportunities to achieve personal and organisational goals. Rapid technological progress in organisations, especially the emerging application of AI technology in the workplace, makes it possible for employees to perceive changes in job characteristics, and change their work behaviours and work outcomes (Virgilio & López, 2021). The development of AI technology and its wide application have brought about significant changes in human work and life. The powerful capabilities of AI in data analysis and statistical predictions enable it to achieve higher accuracy when performing data-intensive tasks in management organisations. Therefore, AI can serve as an auxiliary tool for employee creativity. With its powerful data processing and analysis capabilities, AI can help employees quickly obtain information and optimise decisions, thus improving work efficiency. At the same time, AI can also provide some creative suggestions and solutions, and employees can continue to optimise on this basis to stimulate their own inspiration and creativity, so as to improve employees’ creativity. In addition, the excellent problem-solving and analysis capabilities of AI have been proven in improving the work efficiency of employees, and the improved efficiency makes employees more able to pursue higher work goals, thus showing a higher level of creativity in the work process (Zhang et al., 2025). Based on the universality of the introduction of AI in China, and the high maturity of AI application technology in domestic enterprises, employees are quite accustomed to and have a high acceptance rate of applying AI to their daily work. Therefore, this study believes that the introduction of AI can stimulate work efficiency and promote creativity for employees. The following hypotheses are put forward in this study:

**Hypothesis** **1.** *The introduction of AI will have a significant positive impact on employee creativity.*

### 2.4. Mediating Effect of Perceived Job Autonomy

Theory of Technological Affordances (Gibson, 1977) points out that technology provides the possibility for individuals and organisations to use technology capabilities to pursue better job performance and competitive advantage. As a kind of digital technology, AI has high exhibibility. Employees can obtain visual results and quickly obtain the needed information through the powerful data processing and analysis capabilities of AI. When employees have the autonomy to choose information and data sources and manage and control information flow at work, they have a higher level of perceived autonomy (Oldham & Silva, 2015). In addition, the introduction of AI gives employees the opportunity to add an assistant, that is, “AI”, to help them complete tedious prescribed tasks, so as to free up more time and energy for employees. Employees can arrange their work more flexibly, which brings satisfaction to employees, thus enhancing employees’ work participation and promoting the improvement of perceived autonomy. Therefore, this study assumes that the introduction of AI has a positive effect on employees’ perceived task autonomy.

**Hypothesis** **2.** *The introduction of AI has a significant positive impact on perceived job autonomy.*

The job characteristics theory shows that the use of digital equipment and technology in the work situation will enhance the job autonomy and job feedback in the work (Hackman & Oldham, 1975), and then improve the work participation and focus of employees, thus having a positive impact on the proposal and implementation of innovative ideas. AI is a kind of computer science and technology, in essence, so we expect that the introduction of AI will affect employees’ creativity through perceived job characteristics. Perceived job autonomy refers to whether the job provides employees with sufficient freedom, independence and discretion to arrange working hours and determine working methods. Employee creativity refers to employees’ new ideas about work tasks, processes, and methods generated in their workplace, which will have a beneficial impact on the enterprise in the short term or long term. Hackman and Oldham verified and summarised work outcomes influenced by job characteristics, including job satisfaction, absenteeism rate and turnover rate, job performance and intrinsic job motivation (Hackman & Oldham, 1976). Oldham and Silva proposed that flexible and autonomous work arrangements can enhance employees’ job responsibility and enhance their work engagement (Oldham & Silva, 2015), thus promoting the generation of employees’ innovative ideas. The relaxation of task procedures and rules in work gives employees space to explore new methods and processes. Flexible work procedures encourage employees to think about how to improve the existing work methods and procedures. In order to improve their work efficiency, employees will independently explore how to improve the existing work methods to improve their own performance. In addition, flexible work schedules provide space for employees to explore new methods and to find new problems, and employees can reasonably arrange their work tasks, which provides a foundation for the generation of exploratory ideas. The introduction of AI enhances employees’ flexibility in time arrangement, and makes it possible for employees to explore new methods and processes. Therefore, we believe that perceived job autonomy has a positive impact on employee creativity, and perceived job autonomy plays a mediating role in the relationship between the introduction of AI and employee creativity.

**Hypothesis** **3.** *Perceived job autonomy plays a mediating role in the relationship between AI introduction and employee creativity.*

### 2.5. Mediating Role of Perceived Job Feedback

In the current digital work environment, AI is changing the way enterprises interact with their employees (White, 2019). AI can keep front-line employees always in touch, enabling them to quickly access the expertise and solutions needed to serve customers. This technical support has significantly enhanced the speed and quality of service response. As a valuable asset, AI can provide unique, customised suggestions for different types of knowledge workers. AI systems can recommend personalised marketing techniques based on customer purchase records, provide optimisation suggestions based on engineers’ programming habits, and quickly analyse massive documents to point out the direction of innovation research. This kind of customized, intelligent assistance enables workers in various professional fields to obtain targeted support. At the same time, from the perspective of organisational collaboration, AI promotes the digital connection between employees, documents, information, knowledge, and machines. When an employee proposes an innovative idea, the AI system can intelligently identify colleagues in related fields and promote the collision of ideas across departments. This greatly increases the chances of excellent ideas being seen, and also significantly enhances the possibility of employees receiving valuable feedback. The appropriate use of AI systems in the business operations can indeed effectively improve employees’ perception of work feedback. This improvement is not only reflected in the quantity of feedback, but also in the timeliness and relevance of feedback, so as to optimise the overall work experience and efficiency of employees. Therefore, this study believes that the introduction of AI has a positive impact on employees’ perception of task feedback.

**Hypothesis** **4.** *The introduction of AI has a significant positive impact on the perceived job feedback.*

Perceived job feedback refers to whether employees can receive clear and explicit feedback on their work performance when completing tasks at work. Oldham and Silver suggested that the feedback brought by digital technology provides an opportunity for users to exchange ideas and opinions with each other (Oldham & Silva, 2015). Perceived job feedback, as a characteristic of perceptual work, takes the information exchange or knowledge sharing behaviour among users as the carrier, strengthens the connection among users, and promotes the exchange and collision of ideas from different sources. As a result, it has a positive impact on the generation of innovative ideas. The introduction of AI has expanded the ways and speed of employees to receive feedback from the outside world. When receiving feedback from AI, employees may gradually adjust their behaviour based on the opinions or ideas in the feedback content, which is specifically manifested as continuous and minor improvements in work methods or processes. At the same time, the feedback received by employees will also have an inspirational effect on them, promoting the raising of new questions or the generation of new ideas. Therefore, it is believed that perceived job feedback will positively promote employee creativity, and perceived job feedback plays a mediating role in the relationship between the introduction of AI and employee creativity.

**Hypothesis** **5.** *Perceived job feedback plays a mediating role in the relationship between AI introduction and employee creativity.*

### 2.6. Moderating Effect of Technology Overload

Technology overload refers to the situation where employees are required to perform tasks faster and longer (Tarafdar et al., 2007). It can also cause employees to receive more information than they are able to handle or use (Karr-Wisniewski & Lu, 2010), and exchange more information than they need (Tayyba et al., 2022). The phenomenon of technological overload can be defined as a state of stress arising from digital work demands that exceed an individual’s cognitive resources. This imbalance is characterised by a disparity between ‘demands and capabilities’ (Gimpel et al., 2024; Pflügner et al., 2024). The theory of technological affordances indicates that the use of specific technologies can provide action opportunities for employees to achieve personal and organisational goals, but due to the complex relationship between these technologies and their users, the application of AI technologies in work is often limited by technology. These limitations are manifested in the fact that the powerful data processing and analysis capabilities of AI may increase the work pressure on employees. In the event of employees utilising AI to access information, the volume of feedback provided may far exceed their requirements. Generative AI has been observed to generate a substantial number of redundant options, necessitating manual filtering and consolidation (Boussioux et al., 2024a; Sun et al., 2025). This has led to employees being compelled to sift through vast quantities of data to identify relevant content. This phenomenon has been shown to increase workload, as well as consume time and mental energy processing information, leading to a perceived reduction in employees’ autonomy (Fabienne et al., 2022). Meanwhile, AI will continuously provide employees with work information. This constant connectivity can cause employees to feel uncomfortable with extra work, being ‘on call’ during non-working hours, as well as the resulting anxiety and stress of overwork. In addition, continuous feedback can bring great psychological stress to employees, and this stress and anxiety can cause employees to have less motivation and energy to make their work output more creative (Karr-Wisniewski & Lu, 2010), and employees’ perceived job feedback will be reduced. The phenomenon of technological overload, understood as a source of digital impedance stress, has been demonstrated to induce emotional exhaustion and directly impair work performance and creativity (Pflügner et al., 2024). Therefore, this paper puts forward the following hypotheses:

**Hypothesis** **6.** *Technology overload negatively moderates the relationship between AI introduction and perceived job autonomy, that is, the higher the technology overload is, the weaker the positive relationship between AI introduction and perceived job autonomy is.*

**Hypothesis** **7.** *Technology overload negatively moderates the relationship between AI introduction and perceived job feedback, that is, the higher the technology overload is, the weaker the positive relationship between AI introduction and perceived job feedback is.*

### 2.7. Theoretical Framework

**Figure 1:**
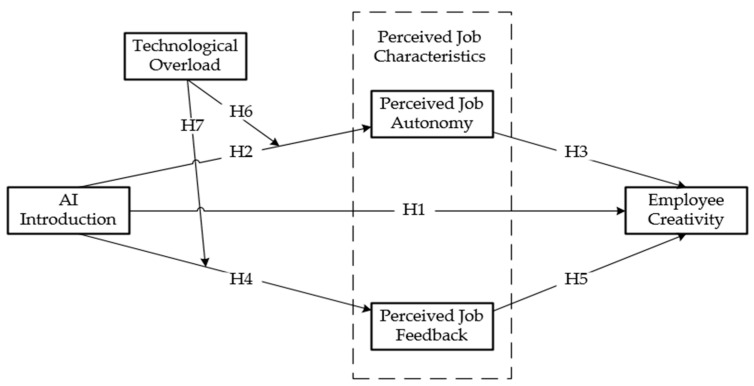
Theoretical model.

The theoretical framework of this paper is shown in Figure 1 below.

## 3. Methodology

### 3.1. Research Methodology and Sample

The present study employed an online survey platform for the purpose of data collection. In light of the inherent limitations of the service’s sample, specifically the inability to communicate directly with respondents, a number of quality control measures were implemented to ensure data integrity (Podsakoff et al., 2003). The questionnaires were designed to impose reasonable time limits, thereby ensuring that respondents would be compelled to complete the survey within a specified timeframe. This approach was adopted to mitigate the risk of respondents providing haphazard or hurried responses. The survey was conducted in two phases, with a 30-day interval between the first and second rounds of data collection, thereby reducing the impact of temporary emotional or situational factors on the results (Dormann & Griffin, 2015).

This study employed an online questionnaire survey method, collecting data in two phases, to examine the impact of introducing AI on employee creativity and its underlying mechanisms. The research subjects comprised full-time employees from multiple organisations across China. The sample was selected using stratified random sampling, stratified by region, industry, and enterprise size, to ensure national representativeness and diversity across organisational contexts. The questionnaire was distributed via a professional online survey platform, with active employees widely invited to participate through multiple channels, including WeChat groups, WeCom, and QQ groups.

Phase 1 (T1): A total of 350 questionnaires were distributed, yielding 332 valid responses. Variables measured in this phase included: demographic variables (gender, age, educational attainment, etc.), degree of AI implementation, and technological overload. Phase Two (T2): Thirty days later, questionnaires were redistributed to T1 participants. After excluding questionable responses and matching the two datasets, 309 valid questionnaires were ultimately obtained, achieving an overall valid matching rate of 88.3%. This phase primarily measured mediating variables (perceived job autonomy, perceived feedback) and outcome variables (employee creativity). To ensure accurate data matching across time points, participants were requested to provide the last four digits of their mobile phone number as an anonymous identifier. All participants voluntarily consented to participate under informed consent, with assurances that their responses would remain confidential and used solely for academic research purposes. The demographic characteristics of the employee sample are shown in Table 1.

### 3.2. Measurement Tools

The scale used in this study is frequently used in mainstream journals, at home and abroad. In this survey, all questionnaires are completed by employees, and the scale used is a seven-point Likert scale with response options ranging from 1 (strongly disagree) to 7 (strongly agree).

AI Introduction scale: A 3-item scale developed by Man Tang et al. was used to investigate the degree of AI application (Tang et al., 2022). A representative item was ‘I spend most of my time dealing with AI.’ The Cronbach’s α coefficient of this scale is 0.887.

Perceived job autonomy scale: A 3-item scale developed by Hackman and Oldham (1975) was used to measure perceived job autonomy. A representative item was ‘The introduction of AI allows me to flexibly decide how to carry out my work.’ The Cronbach’s α coefficient of the scale was 0.843.

Perceived job feedback scale: A 3-item scale developed by Morgeson and Humphrey (2006) was used to measure perceived job feedback. A representative item was ‘The introduction of AI allows me to obtain direct and effective information feedback on my work performance.’ The Cronbach’s α coefficient of the scale is 0.861.

Technology overload scale: A 5-item scale developed by Shu et al. (2011) was used to measure technology overload, with a representative item being ‘Technology forces me to do more work than I can handle.’ The Cronbach α coefficient of this scale is 0.885.

Employee Creativity Scale: A 4-item scale developed by Tierney and Farmer (2002) is used to measure employee creativity, and a representative item is ‘I always take the lead in trying new ideas and methods.’ The Cronbach α coefficient of this scale is 0.884.

Control variables: Referring to the results of previous researchers, five variables were selected as control variables in this study: gender, age, education level, company nature, and working years.

All scales demonstrated good reliability (α > 0.80). Convergent and discriminant validity were examined via confirmatory factor analysis (CFA), with all fit indices meeting acceptable criteria (χ^2^/df < 2, CFI > 0.90, RMSEA < 0.05).

Please refer to the Appendix A for the specific questionnaire items.

### 3.3. Data Analysis Methods

The analysis was conducted using statistical software such as AMOS 24 and SPSS 27. The analytical process comprised the following steps:

Confirmatory Factor Analysis (CFA): CFA was prioritised as the initial analytical step to validate the structural validity of latent variables. Model fit was assessed using three key indices: Comparative Fit Index (CFI), Tucker–Lewis Index (TLI), and Root Mean Square Error of Approximation (RMSEA). This choice aligns with methodological standards proposed by Hair et al., who emphasise that combining incremental fit indices (CFI/TLI ≥ 0.90) with absolute fit indices (RMSEA ≤ 0.08) minimises the risk of misjudging model adequacy(Hair et al., 2018). This is crucial for ensuring psychometric rigour when measuring models in research examining workplace technologies and employee outcomes (Podsakoff et al., 2011).

Descriptive Statistics and Correlation Analysis: Descriptive statistics (means, standard deviations) and Pearson correlation coefficients were calculated to characterise variable distributions and preliminary bivariate relationships. Field notes this step is justified because descriptive statistics establish baseline data characteristics (Field, 2013), while Pearson correlation coefficients—suitable for linear relationships between continuous variable—help identify potential multicollinearity (a critical prerequisite for regression analysis) and refine assumptions about variable associations. For instance, prior to examining mediation effects, correlation analysis confirmed directionally consistent relationships among AI adoption, mediating variables, and employee creativity, as recommended by Baron and Kenny in mediation research designs (Baron & Kenny, 1985).

Stratified Regression Analysis: Stratified regression was selected to examine main effects, mediating effects, and moderating effects—consistent with its application in organisational research to isolate incremental variance explained by predictor variable. Specifically, stepwise variable inclusion enabled rigorous testing of whether AI adoption independently predicts employee creativity (main effect), and whether this relationship is mediated by perceived job characteristics or moderated by technological overload—a methodology validated by Hayes for examining complex causal pathways in social science data (Hayes, 2013).

Simple Slope Analysis: To further elucidate the significant moderation effect, a simple slope analysis was conducted (with moderation effect plots). They emphasise that this method clarifies how the strength/direction of the relationship between the predictor and outcome variables varies across different levels of the moderator variable (e.g., high vs. low technological overload). By comparing the slopes of AI adoption’s impact on perceived job characteristics across the two moderator levels, this analysis provides actionable insights into the boundary conditions of the findings—a crucial step toward advancing theoretical understanding of when AI adoption influences workplace perceptions (Preacher et al., 2006).

Bootstrap Method: This study employed the bootstrap method (5000 resampling iterations, 95% confidence interval [CI]) to test the mediating effect (Preacher & Hayes, 2004). Bootstrapping enables accurate inference of statistical significance by estimating the sampling distribution of indirect effects through resampling from the original datase. This method ensures the validity of conclusions regarding whether perceived job autonomy/feedback mediates the relationship between AI introduction and employee creativity.

## 4. Results

### 4.1. Common Method Bias

The Harman’s Single Factor Test was utilised in this study, and the findings indicated that the variance explained by the first factor prior to rotation was 38.387%, which is less than the threshold of 40%. Furthermore, the variance inflation factor (VIF) was utilised to assess the correlation between the variables. It is generally accepted that a VIF value greater than 5 is indicative of potential multicollinearity. The findings indicate, that when creativity among employees is considered the dependent variable, the VIF values of AI introduction, perceived job autonomy, perceived job feedback and technology overload are 1.113, 3.454, 3.495 and 1.029, respectively, all falling below the 5 threshold. This suggests that multicollinearity is not a significant concern. This finding suggests that the measurement outcomes of this study are not significantly influenced by common method bias.

### 4.2. Confirmatory Factor Analysis

The present study employs confirmatory factor analysis, a statistical technique used to test the reliability and validity of a measurement model using Amos 24 software. The detailed results of this analysis are presented in Table 2. As demonstrated in Table 2, the ratio of Chi-square degrees of freedom is 1.522, which is less than 3. The values of GFI, TLI and IFI are 0.94, 0.978, and 0.983, respectively, all exceeding the threshold of 0.90. The RMSEA value is 0.041, which is below the threshold of 0.05. All the fitting indexes of the five-factor model meet the standard, and the model’s fitting degree is significantly better than that of other models. These findings indicate that these five variables possess high discriminant validity.

### 4.3. Descriptive Statistics and Correlation Coefficient

The results of the correlation analysis of the variables in this study are shown in Table 3. As shown in Table 3, AI introduction is positively correlated with perceived job autonomy (r = 0.271, *p* < 0.01), perceived job feedback (r = 0.292, *p* < 0.01) and employee creativity (r = 0.303, *p* < 0.01). Perceived job autonomy (r = 0.763, *p* < 0.01), and sensory feedback (r = 0.767, *p* < 0.01) and employee creativity are related. These results have initially verified the hypotheses proposed in this study.

### 4.4. Hypothesis Testing

#### 4.4.1. Main Effects Test

This research adopts the hierarchical regression analysis method to analyse the AI is introduced into a direct relationship with the employee creativity. The regression analysis results are shown in Table 4, and Model 1 only includes the regression analysis results of control variables on employee creativity. Model M6 adds AI introduction as a predictor variable on the basis of control variables, and the results show that AI introduction has a significantly positive impact on employee creativity (β = 0.306, *p* < 0.01). This result shows that the higher the degree of AI introduction in enterprises is, the stronger the creativity of employees is. Therefore, Hypothesis 1 is supported.

#### 4.4.2. Mediating Effect Test

Table 4 shows that Model M2 tests the impact of AI introduction on perceived job autonomy, on the basis of control variables. The results show that the introduction of AI has a significantly positive impact on perceived job autonomy (β = 0.267, *p* < 0.01). Hypothesis 2 is proved. Model M7 is on the basis of the M6 with employees’ perceived autonomy in their work variables. The impact of AI introduction on employees’ creativity decreased from 0.306 (*p* < 0.01) to 0.107 (*p* < 0.01), and perceived job autonomy has significant positive effects on employee creativity (β = 0.745, *p* < 0.01). Therefore, perceived job autonomy in the AI introduction and employee creativity plays a role of intermediary between the assumption of H3.

On the basis of control variables, Model M4 examines the impact of AI introduction on perceived job feedback. The results show that the introduction of AI has a significantly positive impact on perceived job feedback (β = 0.292, *p* < 0.01). Hypothesis H4 is verified. Model M8 adds the variable of employees’ perceived job feedback on the basis of M6. The impact of AI introduction on employees’ creativity decreases from 0.306 (*p* < 0.01) to 0.088 (*p* < 0.05), and perceived job feedback has a significantly positive impact on employees’ creativity (β = 0.747, *p* < 0.01). Therefore, perceived work feedback in AI introduction and employee creativity plays a role of intermediary between the assumption of H5.

This study employs the Process 4.1 macro model to examine the parallel mediating effects of perceived job autonomy and perceived job feedback in the relationship between the introduction of AI and employee creativity. Bootstrap sampling was conducted through 5000 iterations with a confidence level of 95%. The results, shown in Table 5, show that both the overall and direct effects of AI introduction on employee creativity are significant, and the confidence intervals do not contain zero. The effect value of the indirect effect of AI introduction on employee creativity through perceived job autonomy is 0.136, and its 95% confidence interval does not contain zero. AI work introduced by sensing feedback indirectly affect employee creativity effect value of 0.150, 95% confidence interval does not include the same zero. Therefore, Hypothesis 3 and Hypothesis 5 are further supported.

#### 4.4.3. Moderating Effect Test

The test results of the moderating effect of technology overload are shown in Table 6. According to the analysis results of Models 9 and 10, it is found that the interaction term of AI introduction and technology overload has a significantly negative impact on perceived job autonomy (β = −0.259, *p* < 0.001), and technology overload negatively moderates the relationship between AI introduction and perceived job autonomy, which confirms H6. According to the analysis results of Models 11 and 12, it was found that the interaction term between AI introduction and technology overload had a significant negative impact on perceived job feedback (β = −0.223, *p* < 0.001), confirming Hypothesis H7.

**Figure 2:**
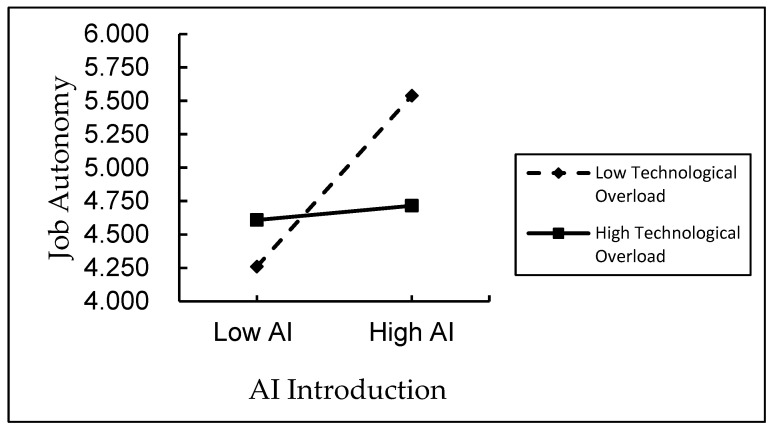
Moderating effect of technology overload on perceived job autonomy.

**Figure 3:**
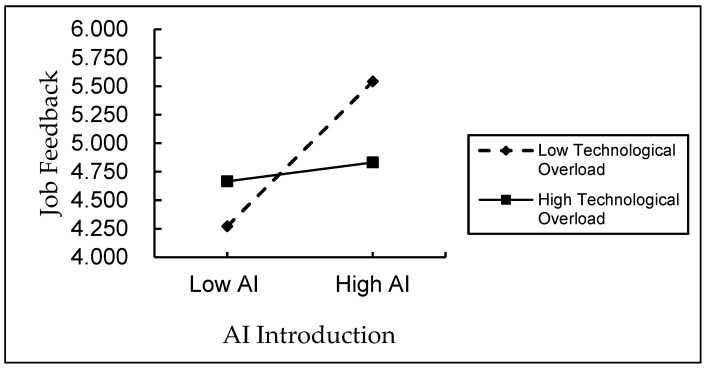
Moderating effect of technology overload on perceived job feedback.

In order to provide further clarification regarding the moderating effect of technology overload on the relationship between AI introduction and perceived job autonomy and perceived job feedback, this study constructs the moderating effect graphs, respectively. Figure 2 demonstrates that, as technology overload increases, the positive relationship between AI introduction and perceived job autonomy becomes less pronounced. Figure 3 demonstrates that, in circumstances of elevated technology overload, the positive relationship between AI introduction and perceived job feedback becomes less pronounced.

### 4.5. Hypothesis Test Results

The present study posited a total of seven research hypotheses. The empirical analysis yielded the test results displayed in Table 7, thereby providing substantiation for all hypotheses.

These findings are closely aligned with the propositions of the theory of affordances (Gibson, 1977), which posits that technological artefacts offer possibilities for action that can reshape user behaviour and cognitive processes. The positive effects of AI introduction on both creativity (H1) and the mediating roles of autonomy and feedback (H3, H5) are consistent with prior studies suggesting that AI can enhance human capabilities by reducing cognitive load and offering new interactive pathways (Hackman & Oldham, 1976).

However, the negative moderating role of technology overload (H6, H7) introduces a critical nuance: when AI systems become overwhelming—either through complexity or excessive demands—they may inhibit rather than enable autonomy and feedback. This finding is consistent with recent literature on the “dark side” of AI adoption, which posits that technology overload can lead to burnout and a reduction in perceived control (Tarafdar et al., 2019). This finding highlights the dual role of AI, as both an enabler and a potential stressor.

The findings of this study corroborate the theoretical model, thereby offering a more nuanced understanding of the manner in which AI influences employee experiences. The results of this study lend support to the affordance perspective, while concomitantly underscoring the boundary conditions that shape its applicability in organisational contexts.

## 5. Discussion

Based on the theory of technological affordances and job characteristics theory, the present study is an attempt to reveal the relationship between the introduction of AI in enterprises and employee creativity, in order to address the research gap. Concurrently, a comprehensive theoretical model is being developed to comprehensively analyse the multiple mediating effects of perceived job autonomy and perceived job feedback, as well as the moderating effect of technology overload. The present study has analysed the survey data from 309 two-time point questionnaires and the following conclusions have been reached.

Firstly, this study found the promoting effect of AI introduction on employee creativity, and revealed the mediating role of perceived job autonomy and perceived job feedback in this process. Introduced specifically, AI can enhance employees’ job autonomy and feedback, thereby increasing their work engagement and concentration, and ultimately having a positive impact on the proposal and implementation of innovative ideas (Hackman & Oldham, 1976). The introduction of AI provides employees with more autonomy. The utilisation of AI tools has the capacity to liberate employees from time-consuming and repetitive tasks, such as data entry and document sorting. This, in turn, enables them to allocate their time and energy towards more creative and valuable endeavours (Oldham & Silva, 2015). Moreover, the implementation of AI has the potential to facilitate the exploration of novel methods and processes by employees. Flexible work procedures have been shown to encourage employees to consider ways in which existing work methods and processes may be improved. In order to enhance their own work efficiency, employees will undertake independent research into the potential for refining existing methods, with a view to improving their own performance (Hackman & Oldham, 1976). This process of independent exploration not only improves employees’ skill levels, but also stimulates their creativity.

Secondly, the integration of AI has led to a substantial augmentation in the accessibility and velocity with which employees can obtain feedback. The utilisation of AI tools has the capacity to facilitate the real-time analysis of employees’ performance, in addition to the provision of immediate feedback (Oldham & Silva, 2015), which can help employees quickly identify their own value and reduce decision-making mistakes caused by information lag. The introduction of AI has also been demonstrated to promote interaction and knowledge sharing among employees. The intelligent collaboration platform facilitates the exchange of ideas and opinions among employees, as well as the sharing of experiential knowledge, thereby enabling the establishment of optimal practices (Oldham & Silva, 2015). This kind of interaction has been shown to enhance the connection between employees and to promote the exchange and collision of ideas from different sources, thus stimulating innovation inspiration.

Finally, technology overload has been shown to exert a negative moderating influence on the relationship between AI introduction and perceived job autonomy and perceived job feedback. Specifically, when employees use AI to obtain information, they must sift through a substantial amount of data to identify the content that is genuinely useful to them. In circumstances where employees are overwhelmed by technology, they will expend considerable energy in the process of information filtering. This will result in a significant decline in their sense of control and autonomy in their work. This process has been shown to increase the workload of staff, as well as occupying their time and energy, thereby reducing the employees’ perceived job autonomy. The utilisation of AI technology facilitates the real-time monitoring and analysis of employees’ work status, thereby enabling the continuous provision of feedback information. Whilst this constant connectivity may improve productivity to a certain extent, it may also cause employees to experience a certain degree of discomfort with the additional workload. In circumstances where technology overload is prevalent and the information provided by AI is both too frequent and lacks specificity, employees may experience a sense of being overwhelmed by information, consequently finding it challenging to extract valuable content. This phenomenon of information overload has been demonstrated to have a detrimental effect on the efficacy of feedback, and may also result in employees exhibiting resistance to the feedback.

### 5.1. Theoretical Implications

Firstly, this study explores the impact of AI introduction on employee creativity and puts forward management innovation suggestions to enhance employee creativity. The research results will provide valuable new findings for management theory innovation. Secondly, it has expanded the literature related to AI and human collaboration. Currently, there are many studies in the academic community on the negative effects of AI on employees, such as job insecurity and job threats. However, research on the positive effects of AI and human collaboration is relatively scarce. This study focuses on strategies for enhancing employee creativity in the era of AI, which will provide references for subsequent research. Thirdly, this study finds that perceived job characteristics play a mediating role between AI introduction and employee creativity, which enriches the related research on perceived job characteristics in the era of AI. Fourth, this study finds that technology overload plays a moderating role between AI introduction and employee creativity, which enriches the related research on technology overload in the era of AI.

The findings of this study will benefit both individuals and organisations. On the one hand, for individuals, studying the impact of the introduction of AI on employee creativity can promote the improvement of employee creativity in the era of AI, and promote their personal career development. The research results also suggest that employees should keep pace with the times, be proficient in the use of AI technology for problem-solving in the workplace, enhance their perceived autonomy and feedback, continuously enhance their skills related to AI collaboration, and aspire to become lifelong learners. Conversely, organisations must direct their undivided attention to the imminent changes in work environments and talent transformation precipitated by AI. It is imperative that organisations adopt an AI-based approach in order to cultivate a corporate culture that fosters employee adoption of change and novel technologies. Furthermore, the formulation of targeted talent development strategies is essential for the purpose of enabling employees to evolve in tandem with AI. Such strategies should be designed to enhance employee creativity and augment the enterprise’s competitive edge. Concurrently, it is imperative to circumvent the deleterious consequences of excessive AI utilisation, thereby ensuring its efficacious service to employees and the stimulation of their creativity. The findings of this study will furnish pertinent, theoretical counsel to managers, thereby facilitating the enhancement of employee creativity in the context of AI integration.

### 5.2. Practical Implications

Organisations have the capacity to develop bespoke training programmes that systematically focus on enhancing three key employee competencies: technology acceptance, creative self-efficacy, and human–machine collaboration capabilities. This enables them to fully leverage the positive impact of AI on employee creativity. In order to achieve this, companies should implement multi-tiered, structured training initiatives that empower employees to effectively boost their innovative performance in increasingly AI-integrated work environments.

The following specific measures have been implemented: Firstly, the implementation of case studies, scenario simulations and value-awareness workshops is recommended, with the objective of enabling employees to develop a profound comprehension of the practical value and application scenarios of AI technology within the context of their specific roles. This has been shown to enhance users’ perception of the usability and accessibility of AI tools, thereby increasing their willingness to use these tools and fostering a proactive attitude towards integrating AI technology into their work. Secondly, there is a necessity to expand the provision of practical training courses with a focus on human–machine collaboration. It is imperative that content encompasses fundamental AI tool operations, data interpretation and analysis skills, as well as the methodology of operating with AI systems to complete complex tasks and collaboratively solve real business problems. Such training should emphasise experiential learning and collaboration, underscoring the pivotal role of humans in decision-making processes while acknowledging the supportive function of AI in enhancing cognition and efficiency. Finally, training should strive to enhance employees’ creative self-efficacy, defined as their confidence in leveraging AI tools to generate innovative outcomes. This objective can be accomplished through the utilisation of case studies that illustrate successful implementations, the attainment of goals that are achieved in a phased manner, and the provision of timely feedback and recognition of innovative achievements. This approach is intended to gradually build and reinforce employees’ confidence in innovating within AI-augmented environments.

Enterprises should align AI applications with employees’ needs for autonomy, innovation, and interactivity. It is imperative to optimise the potential of AI while mitigating its potential adverse effects on human cognition and collaboration through meticulous management design. The ultimate objective is to achieve “AI-powered creativity.” The utilisation of AI facilitates the expeditious filtration of external information, amalgamating it with existing internal knowledge to establish a bespoke innovation framework. This framework assists employees in transcending conventional cognitive boundaries. Enterprises can also utilise AI to provide multi-dimensional feedback from systems, colleagues, and tasks. The regular review of AI-generated interpersonal feedback, including real-time analyses of team contributions, the adoption rates of creative ideas, and cross-functional collaboration, has been demonstrated to provide employees with clarity regarding their value, even in technology-saturated environments.

Furthermore, it is incumbent upon companies to establish systematic safeguards that serve to balance the efficiency of AI with the protection of creative thinking. The real-time evaluation of employees’ AI usage, including frequency, dependency and interruption rates, can facilitate the assessment of technology overload. The utilisation of incentive mechanisms that recognise achievements independent of AI assistance has been demonstrated to elicit a profound motivation in employees to delve more profoundly into the realms of their own capabilities. It is imperative that performance evaluation systems place greater emphasis on individual creativity, critical thinking, and problem-solving ability, as opposed to merely technical proficiency, during promotion assessments.

### 5.3. Research Limitations and Directions

The present study was constrained by limitations in the selection of the sample; as a result, factors such as industry attributes, regional characteristics, and cultural backgrounds were not sufficiently incorporated. Given the marked differences observed across various industries with regard to technological application and work nature, it is to be expected that the impact of AI on creativity will exhibit industry-specific characteristics. It is recommended that future research be conducted in the form of multi-industry, multi-context field investigations and experiments, with a view to enhancing causal inference capabilities through variable control in order to improve the generalisability of conclusions. Moreover, the present reliance on a solitary data source carries risks of common method bias. The utilisation of multi-temporal, multi-source paired data collection methodologies (e.g., leader-subordinate paired questionnaires, peer or superior evaluations) is advocated in order to procure more multidimensional and reliable data.

Whilst the present study focuses on the relationship between perceived job characteristics and technological overload, cross-level variables such as individual differences (e.g., skill levels and technology acceptance) and contextual factors (e.g., organisational support and industry competition) may also influence the association between AI implementation and employee creativity. In view of the intricacies inherent in these factors, it is important to note that the study’s conclusions may not fully reflect actual conditions in certain scenarios. Future research should therefore prioritise incorporating contextual factors such as algorithmic governance and leadership, alongside exploring alternative pathways centred on self-efficacy. The implementation of such a system would facilitate the testing of more sophisticated theoretical models. The employment of multilevel linear models to analyse how individual and organisational factors influence the relationship between AI implementation and creativity aims to provide more targeted recommendations for corporate AI applications.

## 6. Conclusions

This study examines the mechanism through which the introduction of AI influences employee creativity, utilising questionnaire surveys conducted at two distinct time points, and grounded in the theory of technological affordances and Job Characteristics Model. Findings indicate that AI implementation significantly enhances employee creativity by elevating perceived job autonomy and perceived feedback. Concurrently, technological overload exerts a negative moderating effect throughout this process; when employees perceive heightened levels of technological overload, the positive impact of AI on perceived job characteristics is diminished. This research not only provides theoretical support for AI applications in organisational management but also reveals its underlying mechanisms and boundary conditions. When implementing AI technologies, enterprises should prioritise employee perceptions and experiences. Through systematic training and task design to mitigate technological overload, they can maximise AI’s potential to empower employee creativity, thereby achieving organisational innovation goals through human–machine collaboration.

## Figures and Tables

**Table 1:** Descriptive statistics of basic employee information.

Items	Features	Percentage (%)
Gender	male	43.4
	female	56.6
Age	Age 20 and younger	2.9
	Ages 21–30	40.8
	Ages 31–40	48.9
	Ages 41–50	4.9
	Between 51 and 60 years old	2.3
	Over 60	0.3
Education	Junior college	23.9
	Undergraduate	58.9
	Master’s	15.5
	PhD	1.6
Nature of business	Soes/central enterprises	14.2
	Government agencies	6.5
	Private enterprises	57.3
	Foreign enterprises and Sino-foreign joint ventures	7.4
	Other vocational jobs	14.6
Years of employment	Less than one year	16.5
	1–3 years (including 3 years)	24.6
	4–5 years (including 5 years)	16.5
	6–10 years (including 10 years)	31.1
	More than 10 years	11.3

**Table 2:** Confirmatory factor analysis of model fit.

Model	χ^2^/df	GFI	TLI	RMSEA	IFI
Five-factor (AI, JA, JF, EC, TO)	1.522	0.940	0.978	0.041	0.983
Four-factor (AI, JA, JF + EC, TO)	2.318	0.894	0.945	0.065	0.954
Three-factor (AI, JA + JF + EC, TO)	2.375	0.886	0.943	0.067	0.951
Two-factor (AI + JA + JF + EC, TO)	6.319	0.753	0.779	0.131	0.807
Single-factor (AI + JA + JF + EC + TO)	12.345	0.561	0.528	0.192	0.586

Note: AI: AI introduction; JA: perceived job autonomy; JF: perceived job feedback; EC: employee creativity; TO: technology overload; the same below.

**Table 3:** Descriptive analysis of all variables.

Variables	M	SD	1	2	3	4	5	6	7	8	9
1 Gender	1.566	0.496									
2 Age	2.638	0.750	0.004								
3 Education	3.948	0.677	0.059	−0.107							
4 Nature	3.016	1.138	0.087	−0.168 **	−0.041						
5 h	2.961	1.294	−0.011	0.574 **	−0.125 *	−0.191 *					
6 AI	5.348	1.023	−0.017	0.024	−0.075	0.016	0.026				
7 JA	4.853	1.416	0.043	−0.111	−0.080	0.105	−0.027	0.271 **			
8 JC	4.870	1.412	0.087	−0.047	−0.022	0.094	0.022	0.292 **	0.842 **		
9 EC	5.235	1.287	0.056	−0.072	−0.011	0.033	−0.038	0.303 **	0.763 **	0.767 **	
10 TO	4.315	1.357	0.075	0.047	−0.108	0.014	0.101	−0.149 **	−0.111	−0.104	−0.236 **

Note: * *p* < 0.05; ** *p* < 0.01; the same below.

**Table 4:** Results of the hierarchical regression model.

Variables	JA		JF		EC			
	M1	M2	M3	M4	M5	M6	M7	M8
Gender	0.042	0.046	0.082	0.086	0.056	0.061	0.027	−0.004
Age	−0.141	−0.144	−0.083	−0.087	−0.075	−0.079	0.029	−0.014
Education	−0.087	−0.068	−0.021	−0.001	−0.021	0.001	0.051	0.001
Nature	0.086	0.081	0.088	0.082	0.016	0.010	−0.050	−0.051
Hours	0.060	0.057	0.084	0.080	0.006	0.002	−0.041	−0.058
AI		0.267 ***		0.292 ***		0.306 ***	0.107 **	0.088 *
JA							0.745 ***	
JF								0.747 ***
R^2^	0.032	0.103	0.022	0.107	0.009	0.102	0.600	0.600
△R^2^	0.032	0.071	0.022	0.085	0.009	0.093	0.498	0.498
F	1.992	5.757 ***	1.335	6.000 ***	0.557	5.73 ***	64.438 ***	64.553 ***

Note: * *p* < 0.05; ** *p* < 0.01; *** *p* < 0.001; the same below.

**Table 5:** Mediating effect test results.

Action Path	Effect	SE	LLCI	ULCI
Total effect	0.382	0.068	0.247	0.516
Direct effect	0.096	0.045	0.007	0.185
AI-JA-EC	0.136	0.032	0.076	0.202
AI-JF-EC	0.150	0.035	0.083	0.223

**Table 6:** Results of the moderating effect.

Variables	Variable Name	JA		JF	
		M9	M10	M11	M12
Control variables	Gender	0.053	0.039	0.092	0.081
	Age	−0.146	−0.125	−0.088	−0.07
	Education	−0.078	−0.088	−0.009	−0.018
	Nature of business	0.082	0.079	0.084	0.081
	Hours	0.066	0.055	0.088	0.079
Independent variables	AI	0.253 ***	0.22 ***	0.28 ***	0.252 ***
Moderating variables	TO	−0.087	−0.081	−0.076	−0.071
Interaction terms	AI × TO		−0.259 ***		−0.223 ***
R^2^		0.110	0.175	0.112	0.160
△R^2^		0.007	0.065	0.006	0.048
F		5.303 ***	7.952 ***	5.424 ***	7.158 ***

Note: *** means *p* < 0.001.

**Table 7:** Summary of Hypothesis Testing Results.

Hypotheses	Results
**H1.** *The introduction of AI has a significant positive impact on employee creativity.*	Confirmed
**H2.** *The introduction of AI has a significant positive impact on perceived work autonomy.*	Confirmed
**H3.** *Perceived work autonomy plays a mediating role in the relationship between the introduction of AI and employees’ creativity.*	Confirmed
**H4.** *AI introduction has a significant positive impact on perceived work feedback.*	Confirmed
**H5.** *Perceived work feedback mediates in the relationship between AI introduction and employees’ creativity.*	Confirmed
**H6.** *Technology overload negatively moderates the relationship between AI introduction and perceived work autonomy.*	Confirmed
**H7.** *Technology overload negatively moderates the relationship between AI introduction and perceived work feedback.*	Confirmed

## Data Availability

The data presented in this study are available on request from the corresponding authors.

## Appendix A

**Phase 1**

Basic Information

Your gender: [Single-choice question] *

○Male ○Female

Your age group: [Single-choice question] *

○20 years old and below ○21–30 years old ○31–40 ○41–50 years old

○51–60 years old ○60 years old and above

Education Level [Single Choice] *

○Junior high school or below

○High School/Vocational School/Technical School

○Junior college

○Bachelor’s Degree

○Master’s Degree

○Doctorate

Enterprise Nature [Single Choice] *

○State-Owned Enterprise/Central State-Owned Enterprise

○Government Agency

○Private Enterprise

○Foreign-Owned and Sino-Foreign Joint Ventures

○Other Professional Occupations

Years of Work Experience [Single Choice] *

○Less than 1 year

○1–3 years (including 3 years)

○4–5 years (including 5 years)

○6–10 years (including 10 years)

○Over 10 years

Last four digits of mobile number [Fill-in-the-blank] *

_________________________________

AI adoption [Matrix scale question] *

	**Strongly Disagree**	**Disagree**	**Somewhat Disagree**	**Neutral**	**Somewhat Agree**	**Agree**	**Strongly Agree**
I use artificial intelligence to perform most of my job functions.	○	○	○	○	○	○	○
I spend most of my time interacting with AI.	○	○	○	○	○	○	○
I interact with AI when making significant work decisions.	○	○	○	○	○	○	○

Technology overload [Matrix scale item] *

	**Strongly Disagree**	**Disagree**	**Somewhat Disagree**	**Somewhat**	**Somewhat Agree**	**Agree**	**Strongly Agree**
Due to increased technical complexity, my workload has grown.	○	○	○	○	○	○	○
Technology forces me to work under very tight deadlines.	○	○	○	○	○	○	○
I am forced to change my work habits to adapt to new technology.	○	○	○	○	○	○	○
Technology forces me to take on more work than I can handle.	○	○	○	○	○	○	○
Technology forces me to work faster.	○	○	○	○	○	○	○

**Phase 2**

Perceived job autonomy [Matrix scale question] *

	**Strongly Disagree**	**Disagree**	**Somewhat Disagree**	**Neutral**	**Somewhat Agree**	**Agree**	**Strongly Agree**
The introduction of AI allows me to flexibly decide how to carry out my work.	○	○	○	○	○	○	○
The introduction of AI allows me to flexibly decide when to carry out my work.	○	○	○	○	○	○	○
The introduction of AI allows me to flexibly decide the steps for carrying out work.	○	○	○	○	○	○	○

Perceived Work Feedback [Matrix Scale Item] *

	**Strongly Disagree**	**Disagree**	**Somewhat Disagree**	**Neutral**	**Somewhat Agree**	**Agree**	**Strongly Agree**
The introduction of AI has enabled me to receive constructive feedback on my work from AI.	○	○	○	○	○	○	○
The introduction of AI has enabled me to receive immediate feedback on my performance after completing a task.	○	○	○	○	○	○	○
The introduction of AI has enabled me to receive direct and effective information feedback on my personal work performance.	○	○	○	○	○	○	○

TEmployee Creativity [Matrix scale item] *

	**Strongly Disagree**	**Disagree**	**Somewhat Disagree**	**Neutral**	**Somewhat Agree**	**Agree**	**Strongly Agree**
I always take the lead in trying new ideas and approaches.	○	○	○	○	○	○	○
I always explore new ways to solve problems.	○	○	○	○	○	○	○
I frequently generate breakthrough ideas relevant to this field.	○	○	○	○	○	○	○
I am a good role model for creativity.	○	○	○	○	○	○	○

Last four digits of mobile number [Fill-in-the-blank] *

_________________________________
